# Anxiety, worry and cognitive risk estimate in relation to protective behaviors during the 2009 influenza A/H1N1 pandemic in Hong Kong: ten cross-sectional surveys

**DOI:** 10.1186/1471-2334-14-169

**Published:** 2014-03-27

**Authors:** Qiuyan Liao, Benjamin J Cowling, Wendy WT Lam, Diane MW Ng, Richard Fielding

**Affiliations:** 1 Division of Behavioural Sciences, School of Public health, The University of Hong Kong, 21 Sassoon Road, Pokfulam, Hong Kong, Special Administrative Region, China; 2 Division of Epidemiology and Biostatistics, School of Public Health, The University of Hong Kong, 21 Sassoon Road, Pokfulam, Hong Kong, Special Administrative Region, China

**Keywords:** Influenza pandemic, Affect, Perceived risk, Protective behavior, Chinese adults

## Abstract

**Background:**

Few studies have investigated associations between psychological and behavioral indices throughout a major epidemic. This study was aimed to compare the strength of associations between different cognitive and affective measures of risk and self-reported protective behaviors in a series of ten cross-sectional surveys conducted throughout the first wave of influenza A/H1N1 pandemic.

**Methods:**

All surveys were conducted using questionnaire-based telephone interviews, with random digit dialing to recruit adults from the general population. Measures of anxiety and worry (affective) and perceived risk (cognitive) regarding A/H1N1 were made in 10 serial surveys. Multivariate logistic regression models were used to estimate the cognitive/affective-behavioral associations in each survey while multilevel logistic models were conducted to estimate the average effects of each cognitive/affective measure on adoption of protective behaviors throughout the ten surveys.

**Results:**

Excepting state anxiety, other affective measures including “anticipated worry”, “experienced worry” and “current worry” specific to A/H1N1 risk were consistently and strongly associated with adoption of protective behaviors across different survey periods. However, the cognitive-behavioral associations were weaker and inconsistent across the ten surveys. Perceived A/H1N1 severity relative to SARS had stronger associations with adoption of protective behaviors in the late epidemic periods than in the early epidemic periods.

**Conclusion:**

Risk-specific worries appear to be significantly associated with the adoption of protective behaviors at different epidemic stages, whereas cognitive measures may become more important in understanding people’s behavioral responses later in epidemics. Future epidemic-related psycho-behavioral research should include more affective-loaded measures of risk.

## Background

Understanding relationships between psychological state and protective behaviors during respiratory infectious disease epidemics (RIDEs) can inform risk communication and interventions addressing behavior change [1,2]. Studies of behavioral change during RIDEs usually assess risk perception as an affect-neutral cognitive (“cognitive”) process, commonly using measures such as perceived personal probability of infection or perceived severity of the illness [3-5], or as a more affect-active process, by assessing worry and anxiety [6-10]. The latter are frequently referred to as “affective” dimensions of risk, though worry is often considered a cognitive dimension of anxiety [11]. The dual-process theory proposes that responses to external stimuli involve two different processing systems, one being deliberate, slow and rule-based, the other being experiential, quick and intuitive [12]. These two systems may reflect distinct response pathways to risk: risk-as-analysis (cognitive estimates) and risk-as-feeling (affective estimates) [13]. The affect heuristics and risk-as-feeling hypotheses imply that affect quickly and more efficiently guides cognitive risk analysis and behavior [13-15]. Previous studies found that in the RIDE situation when personal threat is highly uncertain, affective measures of risk more powerfully predict protective behavior uptake than do cognitive measures [6,10]. Therefore, both cognitive and affective components of risk appear to be relevant to understanding RIDEs-related population behavior [1].

In the early epidemic stage when uncertainty about the epidemic characteristics, treatment and prevention is higher, affective responses may be optimal for guiding behavioral change [6,9,10] but cognitive risk responses should increasingly drive behavior as the epidemic evolves. We term these “psycho-behavioural” associations. Given this, the question arises: should studies or assessments done early in the epidemic emphasize affect-based assessments of risk, whereas those performed later in the epidemic emphasize cognitive-based measures, in order to optimally predict behaviors? Otherwise, it is possible that research conducted in different stages of an epidemic may observe different strengths for the same psycho-behavioral association and misattribute these. Observed variation in the strength of specific psycho-behavioral associations across an epidemic introduces avoidable measurement error in the target cognitive/affective measure which will subsequently influence its association with behavioral change, reducing the apparent reliability of risk assessments as predictors of behavior change during RIDEs. This raises questions about whether the same or similar associations would be repeatedly identified in surveys conducted in different epidemic periods within the same population. However, very few studies appear to have examined the consistency of these psycho-behavioral associations across different RIDE stages [6]. We therefore performed secondary data analyses for data collected in a series of ten consecutive cross-sectional surveys spanning the epidemic wave of 2009 pandemic influenza A/H1N1 in Hong Kong [16]. The objectives of this study were to compare the strength and stability of associations between affective and cognitive measures of risk and the adoption of RIDE-related health protective behaviors. This was assessed by comparing the associations between health protective behaviors against A/H1N1 and different cognitive/affective measures of risk used for each of the ten cross-sectional surveys.

Most psycho-behavioral studies of new communicable respiratory disease outbreaks were rapidly implemented [2]. Consequently, many used unrefined questionnaires, with several suffering from minimal theoretical support for the inclusion of specific psychological variables, items of limited utility in understanding behavioral change or items that may have posed task difficulty for respondents [1,2], and multiple items, which increase interview load, thereby reducing interview efficiency and the accuracy of results. To inform future item selection, we therefore also sought to assess the difficulty respondents faced in answering different question measures of risk perception. This was done by examining proportions of missing data for different psychological measures as an indirect reflection of task difficulty.

Our null hypotheses were:

1. Cognitive and affective measures of risk will not differ in terms of stability of association with adoption of protective behaviors across the ten cross-sectional surveys;

2. For the same associations measured at different epidemic periods, strength of associations between affective/cognitive measures and adoption of health protective behaviors will not decline/increase across epidemic stage;

3. There will be no difference in proportions of missing data for cognitive estimation items such as estimates of the likelihood of contracting influenza infection than other risk assessment formats reflecting no differences in the difficulties posed to respondents by such items [17,18].

## Methods

### Data sources

Between April and November 2009, we monitored population psycho-behavioural responses to the 2009 influenza A/H1N1 pandemic using 13 cross-sectional surveys (S1-S13) covering the entire first wave of the A/H1N1 pandemic in Hong Kong [16]. During the survey period, approximately 15% of the Hong Kong population were infected with this new virus [19]. Here we report data from 10 of these (S3-S5 and S7-S13). The first two surveys (S1 and S2) conducted between April and May were excluded from this study because of incompatibility with later surveys and because the local A/H1N1 epidemic did not start until S3 was conducted. Survey S6 was excluded because of sample insufficiency. All surveys utilized identical methods involving random household telephone interviews based on randomly computer-generated landline telephone numbers of all Hong Kong households. One adult aged 18 or above within each household was randomly selected based on a Kish Grid and invited for the telephone interview. Sampling details have been published elsewhere [16]. The study received ethical approval from the Institutional Review Board (IRB) of the University of Hong Kong. The IRB waived written informed consent in lieu of verbal consent given the format of these ten telephone surveys. All participating respondents gave verbal consent for telephone interviews.

The sample sizes for each of the ten surveys (S3-S5, S7-S13) ranged between 1,000-1,404, with response rates of 65.6%-72.7% [16]. Surveys were conducted every two weeks with data collection completed within 3-5 days for each survey. The ten surveys covered different A/H1N1 epidemic stages in Hong Kong. Specifically, S3 (Jun 9-12, 2009) was conducted when local A/H1N1 human cases were first identified in Hong Kong; S10 (Sep 8-11, 2009) was conducted when the local epidemic reached peak activity and S13 (Nov 9-13, 2009) when epidemic activity had declined substantially (Additional file 1: Figure S1).

Core items for the questionnaires used in the ten surveys were retained throughout. Minor changes were made on one measure of risk perception (the comparator “perceived relativesusceptibility relative to others” was made more precise by specifying age and gender at S11) and two new items (current worry and infectivity relative to seasonal flu) were added in later surveys to refine measurement and during the surveys. Table 1 details psychological measures associated with risk covered by different survey periods. Four measures (state anxiety, anticipated worry, experienced worry and current worry about A/H1N1 infection) were classified as affective measures. Four other measures (perceived absolute susceptibility and perceived relative susceptibility to A/H1N1 infection, perceived A/H1N1 severity relative to SARS and perceived A/H1N1 infectivity relative to seasonal influenza) were classified as cognitive estimates of risk. The definitions, questions and response scales for these measures are detailed below and in the Additional file 2: Table S1.

**Table 1:** Psychological measures and their proportions of missing data throughout the 10 surveys

**Measures**	**S3**	**S4**	**S5**	**S7**	**S8**	**S9**	**S10**	**S11**	**S12**	**S13**	**Missing rang%**^**c**^	**Totally missing%**
Anxiety^a^	√	√	√	√	√	√	√	√	√	√	0.20–1.29	0.65
Anticipated worry	√	√	√	√	√	√	√	√	√	√	0.10–1.41	0.59
Experienced worry	√	√	√	√	√	√	√	√	√	√	0–0.21	0.13
Current worry						√	√	√	√	√	0.50–1.12	0.73
Perceived absolute susceptibility	√	√	√	√	√	√	√	√	√	√	1.02–6.63	5.63
Perceived relative susceptibility (perceived personal likelihood of A/H1N1 infection relative to a general person)^b^	√	√	√	√	√	√	√				5.08–7.41	5.82
Perceived relative susceptibility (Perceived personal likelihood of A/H1N1 infection relative to a general person of similar age and gender)^b^								√	√	√	5.17–5.94	5.44
Perceived A/H1N1 severity relative to SARS	√	√	√	√	√	√	√	√	√	√	0.60–2.40	1.50
Perceived A/H1N1 infectivity relative to seasonal flu						√	√	√	√	√	1.93–4.13	3.04

*Note* “√” indicates that the measure was covered in the survey.

^a^The measure of anxiety state included ten items asking about ten general feeling statements and thereby the proportions of missing data for anxiety in the table were the highest proportion of missing data of the item among the ten statement items.

^b^These two items were combined as “perceived relative susceptibility” in the analysis.

^c^The range of missing proportions across the covered surveys.

#### Anxiety

Respondents’ anxiety level was assessed with a previously validated state-anxiety scale of the State-Trait Anxiety Inventory (STAI) wherein respondents’ rate their feelings in response to ten general statements [6,20]. Positive feeling statements were reversely coded and then the mean scores of the ten items (possible range 1 - 4) were calculated for subsequent analyses to overcome the problems of randomly missing items. (Additional file 2: Table S1).

#### Anticipated worry about A/H1N1

Respondents were asked to rate their worry about possibly developing A/H1N1 symptoms within the next 24 hours (Additional file 2: Table S1). Hence this measure was prospective.

#### Experienced worry about A/H1N1

Respondents were asked to recall whether they had experienced any worry over the past week about contracting A/H1N1 (Additional file 2: Table S1). This measure was retrospective.

#### Current worry about A/H1N1

Starting from Survey 9 (Table 1) respondents were asked about their current level of worry related to A/H1N1 (Additional file 2: Table S1). This measure was current.

#### Perceived absolute susceptibility to A/H1N1

Respondents estimated their personal likelihood of contracting A/H1N1 in the coming months throughout the ten surveys (Additional file 2: Table S1). This measure was prospective.

#### Perceived relative susceptibility to A/H1N1

In the earlier surveys, respondents estimated their personal likelihood of contracting A/H1N1 relative to another (unspecified) person in the general population. In later surveys (S11-S13), this item was slightly changed to personal likelihood of contracting A/H1N1 relative to another person *of similar age and sex* in the general population (Additional file 2: Table S1).

#### Perceived A/H1N1 severity relative to SARS

Throughout the 10 selected surveys, respondents estimated the perceived severity of A/H1N1 infection relative to SARS (Additional file 2: Table S1).

#### Perceived A/H1N1 infectivity relative to seasonal influenza

Starting from Survey 9 (Table 1), this item was added in the surveys to assess the infectivity rate of A/H1N1 relative to seasonal influenza, serving as an additional measure to assess the perceived severity of A/H1N1.

The frequencies of three protective behaviors against A/H1N1 were polled throughout the ten surveys. These were avoiding crowded places, maintaining good indoor ventilation and disinfecting the household frequently. All three protective measures were recommended by the Hong Kong government to minimize the transmission of influenza during the epidemic [21]. Respondents were asked whether they had adopted any of these three protective behaviors over the past seven days, and if so, whether the behaviors were adopted for A/H1N1 prevention. These behavioral outcomes were dichotomized as “1” (adopted for preventing A/H1N1) and “0” (not adopted or adopted for reasons other than preventing A/H1N1) for subsequent analyses.

Previous analyses showed trends for psycho-behavioral associations were similar across the responses range on all the above risk measures [16]. Therefore, these responses were dichotomized as either above or below a threshold for subsequent analyses in order to facilitate comparison, and the process detailed in the Additional file 2: Table S1.

### Data analysis

First, the proportions of missing data for all psychological measures associated with risk were calculated. Then, multiple imputation was used to generate ten values for each missing value, the mean of which was substituted for the missing value. For each survey, one multiple logistic regression model calculated the associations between each of three protective behaviors (avoiding crowded places, maintaining good indoor ventilation and disinfecting household frequently) and each psychological variable (psycho-behavioral association) plus the corresponding 95% confidence interval. The psycho-behavioral association was adjusted for respondents’ age, gender, education, marital status and birth place in each logistic regression model because all these demographics are potential confounders of these psycho-behavioral associations [2].

I^2^ (an index of variability) based on Q-statistic was calculated to quantify heterogeneity of these psycho-behavioral associations across the ten surveys and to determine the appropriateness of combining the data from ten surveys to calculate averaged effects. I^2^ produces values ranging between 0 and 100%, indicating the percentage of the total variation across surveys due to heterogeneity rather than chance [22]. Values of 25%, 50% and 75% arbitrarily indicate low, medium and high heterogeneity, respectively [22]. All studied psycho-behavioral associations had either low or low-medium heterogeneity except that the associations between experienced worry and disinfecting the household frequently and between perceived severity relative to SARS and disinfecting the household frequently had medium-to-high heterogeneity across the ten surveys. Therefore, random-effect multilevel logistic regression models were used to estimate the pooled effect of each psycho-behavioral association across the ten surveys. For these multilevel models, individual responses were specified as the first level while survey periods were specified as the second level. All multilevel logistic regression models were adjusted for age, gender, education, marital status and place of birth. To minimize potential interactions (moderation or mediation) between different psychological measures [10,23], only one psycho-behavioral association was assessed in each model.

All analyses were conducted based on data excluding the small proportions (0.2%-1.5%) of subjects who reported having had influenza-like illness (ILI: fever plus cough or sore throat) within the two weeks prior to each survey. P-values <0.05 were considered to be statistically significant. All analyses were conducted using STATA software (version 10.1; STATA Corp., College Station, TX).

## Results

The ten surveys included a total of 10,345 subjects after excluding 92 (0.9%) subjects with ILI. The effect sizes for differences between the sample characteristics (age, gender, education and place of birth) of each survey and the Hong Kong population were small, indicating good sample representativeness [16].

### Missing data for the risk-related psychological measures

Table 1 reports the proportions of missing data for each psychological measure and survey. Among all the measures, perceived absolute susceptibility and perceived relative susceptibility to A/H1N1 infection had the highest proportions (totally missing 5.63% and 5.82%, respectively) of missing data throughout the surveys, followed by perceived A/H1N1 infectivity relative to seasonal influenza (3.04%) and perceived A/H1N1 severity relative to SARS (1.50%). Affective measures generally had few missing data (below 1%, Table 1).

### Psycho-behavioral associations across different A/H1N1 epidemic periods

**Figure 1:**
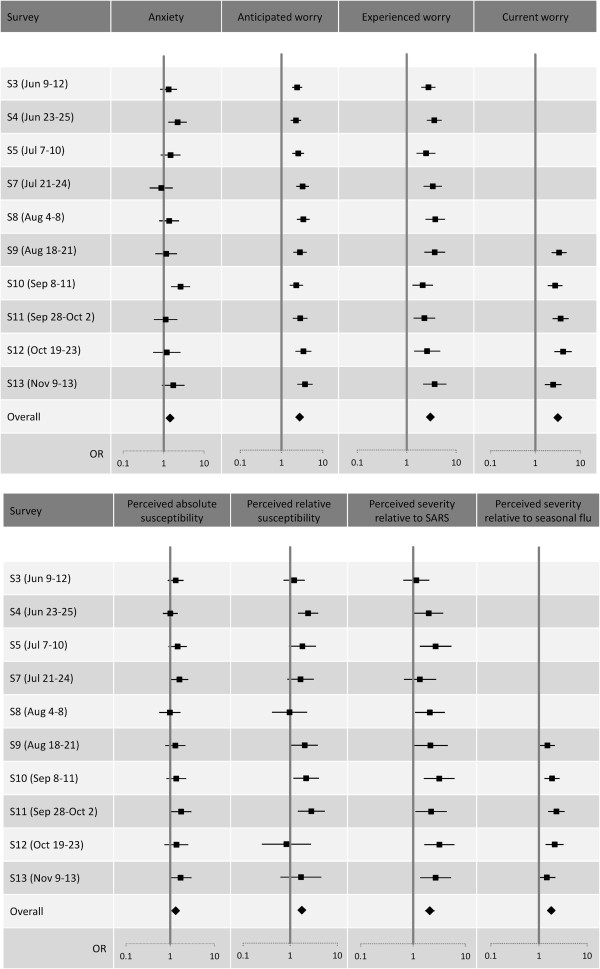
Associations between psychological responses and avoiding crowds during A/H1N1 pandemic.

**Figure 2:**
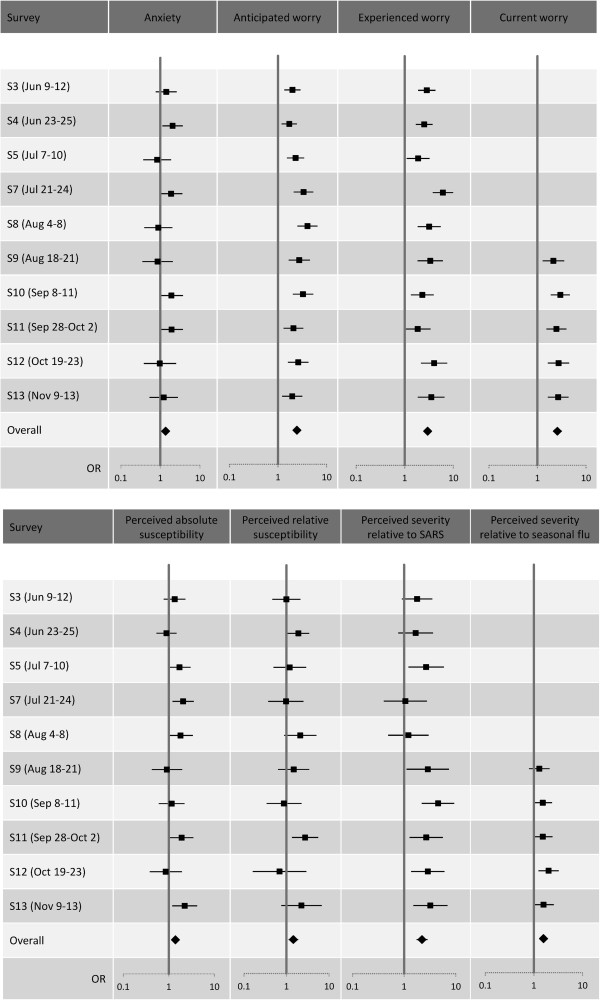
Associations between psychological responses and maintaining good indoor ventilation during A/H1N1 pandemic.

**Figure 3:**
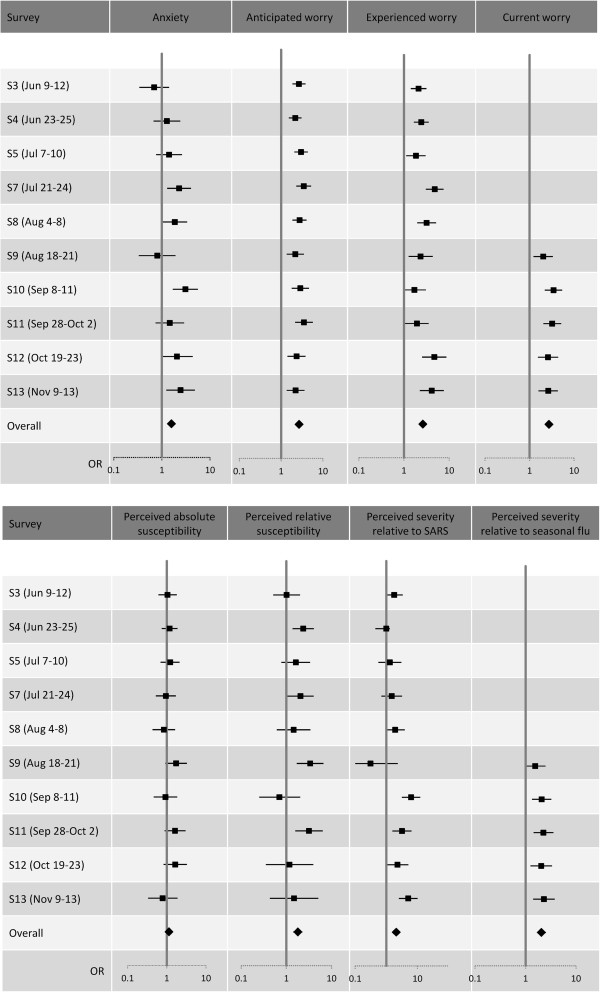
Associations between psychological responses and disinfecting household frequently during A/H1N1 pandemic.

Figures 1, 2 and 3 show forest plots describing the associations of different risk-related psychological measures with avoiding crowded places (Figure 1), maintaining good indoor ventilation (Figure 2) and household disinfection (Figure 3), respectively, throughout the ten surveys. The patterns of psycho-behavior associations were similar for the three types of health protective behaviors. For each of the three figures, the upper four forest plots illustrate the associations between affective measures and adoption of protective behaviors while the lower four illustrate the associations between cognitive measures and adoption of protective behaviors. Averaged effects of different perceptions on adoption of each of the three protective behaviors are indicated by the lower diamond of each forest plot and in Table 2.

**Table 2:** The averaged psycho-behavioural associations across the ten surveys during A/H1N1 pandemic

**Independent variables**	**Avoiding crowded places**	**Maintaining good indoor ventilation**	**Disinfecting household frequently**
Anxiety (high: mean score between 2.5–4.0)	1.44 (1.21–1.73)^***^	1.36 (1.09–1.71)^**^	1.58 (1.29–1.93)^***^
Anticipated worry (more/much more than normal or extremely)	2.75 (2.46–3.07)^***^	2.45 (2.13–2.81)^***^	2.69 (2.36–3.07)^***^
Experienced worry (worry a bit/a lot/all the time)	3.00 (2.62–3.43)^***^	2.96 (2.52–3.47)^***^	2.62 (2.25–3.06)^***^
Current worry (level 6–10)	3.16 (2.64–3.78)^***^	2.57 (2.07–3.18)^***^	2.74 (2.22–3.39)^***^
Perceived absolute susceptibility (likely/very likely/certain)	1.33 (1.14–1.55)^***^	1.40 (1.16–1.70)^***^	1.13 (0.93–1.36)
Perceived relative susceptibility (more/much more/certain)	1.77 (1.44–2.17)^***^	1.45 (1.12–1.88)^**^	1.79 (1.42–2.25)^***^
Perceived severity relative to SARS (severer than SARS)	2.08 (1.69–2.57)^***^	2.22 (1.74–2.83)^***^	2.09 (1.65–2.64)^***^
Perceived severity relative to seasonal flu (severer than seasonal flu)	1.80 (1.51–2.13)^***^	1.58 (1.28–1.95)^***^	2.06 (1.68–2.53)^***^

All data represent odds ratios and their corresponding 95% confidence intervals (in parentheses).

All odds ratios were adjusted by age, gender, education, marital status and birth place.

**p<0.01; ^***^p < 0.001.

Overall, all risk-related psychological variables were positively and significantly associated with all three heath protective behaviors except for the association between perceived absolute susceptibility and household disinfection (Table 2).

Figures 1, 2 and 3 suggest that all affective measures excepting state anxiety are more strongly associated with adoption of protective behaviors than are cognitive measures, these associations being consistently positive and statistically significant across the ten surveys. In particular, current worry and experienced worry had the strongest associations with adoption of protective behaviors among the eight risk-related psychological measures. State anxiety was only significantly associated with avoiding crowds in S4 and S10 (Figure 1), with maintaining good indoor ventilation in S4 (Figure 2), and with household disinfection in S7, S8, S10 and S13 (Figure 3).

Perceived absolute susceptibility was only weakly and significantly associated with avoiding crowds in S7 and S11 (Figure 1) and maintaining good indoor ventilation in S7, S11 and S13 (Figure 2) but not with household disinfection across the ten surveys (Figure 3). Perceived relative susceptibility seemed to have stronger associations with avoiding crowds and household disinfection than did perceived absolute susceptibility (Figures 1 and 2). No change was seen in associations between perceived relative susceptibility compared to another person, and adoption of protective behaviors in S10-S13 when the refined measure of perceived relative susceptibility specified “a general person of similar age and gender”. Perceived higher A/H1N1 severity relative to SARS was more likely to be significantly associated with adoption of protective behaviors in later (S10-S13) than earlier surveys, a pattern not found for other cognitive measures (Figures 1, 2 and 3). Perceived A/H1N1 infectivity relative to seasonal influenza was generally significantly associated with adoption of health protective behaviors but the associations were relative weaker than the associations between perceived A/H1N1 severity relative to SARS and adoption of health protective behaviors (Figures 1, 2 and 3).

## Discussion and conclusions

Our findings were mostly consistent with those hypothesized and the null hypotheses were largely rejected. The main finding is that affective measures of risk perception generally had stronger associations with reported adoption of health protective behaviors during the A/H1N1 pandemic than did cognitive measures. This finding is consistent with those from other studies conducted during both SARS [6] and pandemic A/H1N1 [10,24], suggesting that affective components contribute significantly to adoption of protective behaviors in response to the threat during epidemics over and above simpler cognitive risk estimates. While previous studies were mainly conducted in early epidemic periods [10,24], this study examined affective-behavioral associations across the entire epidemic wave of A/H1N1 in Hong Kong and found that the association between affect-loaded risk measures and adoption of protective behaviors were consistently strong and positive across different epidemic periods.

Studies of the anxiety-behavior association throughout the SARS epidemic found consistently significant and positive associations during the early epidemic phase surveys but mostly non-significant associations in late epidemic phase surveys [6]. The present study did not duplicate this pattern for any of the four affective measures. Reported anxiety level was inconsistently associated with adoption of health protective behaviors in these 10 surveys. One possible reason could be that the measure we used assessed general anxiety only rather than anxiety specific for A/H1N1. Furthermore, overall reported state anxiety levels remained quite stable and consistently low throughout the A/H1N1 epidemic [16], indicating a floor effect, suggesting that a low level of anxiety has little effect on these behaviors. Other affective measures including anticipated worry, experienced worry and current worry generally involve less intense affective components compared with anxiety and thereby are more likely to covary with behavioral change. In particular, our study found that experienced worry and current worry seemed to have stronger associations with adoption of protective behaviors than did anticipated worry. One possible reason could be that the actual affective experience or associated processing may be more strongly associated with behavioral change than its anticipation, which may be subject to forecasting errors [23].

Cognitive risk assessments, in particular perceived susceptibility to A/H1N1 (either absolute or relative susceptibility) had weak associations with adoption of protective behaviors. This suggests that cognitive-behavioral models such as the Health Belief Model [25,26] that rely primarily on purely cognitive estimates of risk to predict behavioral change should perform relatively more poorly at predicting the adoption of protective behaviors during RIDEs. Cognitive-behavioral models generally assume rational processing of external information to inform action. However, during RIDEs particularly in the early stages, uncertainty is usually widespread and poses high [9] or ambiguous personal threat. Consequently, people may face difficulties when attempting to quantify the probabilities of their risk of acquiring the infection and the severity of associated disease. Whether it is threat ambiguity, task difficulty in determining risk magnitude or a primary affective response that modifies cognition, that leads to affect-related measures dominating remains unclear. This study found that the proportions of missing data for purer cognitive risk perception measures, particularly perceived absolute/relative susceptibility to A/H1N1 were greater than for affect-loaded measures, suggesting that respondents may face greater task difficulties in comprehension and/or responses to such questions under epidemic circumstances. Further study is needed to confirm the extent of this effect.

Perceived relative susceptibility seemed to have stronger associations with adoption of protective behaviors than perceived absolute susceptibility. Perceived susceptibility measured in this relative way involves social comparison and accommodates the influences of optimistic bias [27] and therefore probably involves more cognitive processing. More cognitive processing is associated with greater risk estimates and psychological distress [28]. This might account for the more substantial associations with behavioral change than seen for simple personal risk estimates.

Associations between cognitive risk perception measures and protective action were quite inconsistent across the ten selected surveys in this study. Previous reviews concluded associations between cognitive risk perception and adoption of protective behaviors during RIDEs were inconsistent [29]. Our evidence suggests a major reason for this inconsistency lies in these studies being conducted in different epidemic stages [6,30,31]. Our hypothesis was that cognitive factors were more important in changing human behaviors in the later epidemic stage when people had more knowledge and less uncertainty about the threat. This study found that the associations between perceived A/H1N1 severity relative to SARS and adoption of each of the three protective behaviors became significantly and consistently positive starting from survey 10 after the A/H1N1 case confirmations had peaked, consistent with our hypothesis. However, this pattern of associations was not found for perceived susceptibility.

Perceived A/H1N1 infectivity relative to seasonal flu, though not measured before survey S9 had weaker associations with adoption of health protective behaviors, than did perceived A/H1N1 severity relative to SARS in each survey and overall. However, these two measures assessed different aspects of A/H1N1 severity with the former focused on the infectivity rate of A/H1N1 while the latter may primarily focus on the fatality rate of A/H1N1. Further study is needed to confirm which aspects of disease severity could be more important in motivating behavior change.

Study limitations include the serial cross-sectional design and thereby reverse-causality remains a possible explanation. Nonetheless, it is difficult to think of plausible mechanisms whereby, for example, disinfecting one’s home will lead to greater worry regarding infection. Alternatively, the associations could be spurious but this is unlikely given the consistent pattern of the associations in 10 separate samples. It therefore seems most likely that the protective behaviors are consequential on the risk perceptions, and not vice versa. Examining psycho-behavioral associations using longitudinal data during RIDEs is difficult due to their often-rapid evolution and the short lead-time compared to the need to obtain and retain large cohorts for follow-up surveys. Conducting a series of consecutive cross-sectional surveys to investigate the psycho-behavioral associations is a better option than using a single cross-sectional survey. There may be concerns about the generalizability of our findings to more severe RIDEs. For example, during the initial phase of the SARS epidemic, population state anxiety regarding the epidemic was much higher and thereby had strong association with protective behavioral change [6]. However, SARS was the first of the new wave of RIDEs, and a degree of risk fatigue may have subsequently set in. Considering the common situation during RIDEs, we believe that most of the findings in this study could be applicable in other RIDEs. Finally, because all data were self-reported the results may reflect social desirability bias.

This study raises important implications for future respiratory communicable disease-related psycho-behavioral research and public health interventions. First, affective responses improve understanding of behavioral responses throughout different RIDE periods and must form part of measures in relevant studies. However, intense but nonspecific affect such as generalized state anxiety is probably less useful for understanding public behavioral responses during most epidemics where perceived milder threat fails to arouse such affect. Less intense, specific affective responses to a identifiable, if uncertain threat that currently activates or has in the past activated worry may be more likely to show strong and consistent effects on behavioral change across different epidemic periods. Second, cognitive risk estimates during the early epidemic stage may be poor at predicting human behavioral change and present task difficulties to respondents. However, cognitive risk estimates may inform individual behavioral change later in the RIDE epidemic trajectory and should be included in studies conducted during these phases. Relative measures of perceived susceptibility appear superior to perceived absolute susceptibility in predicting behavioral change and thereby are preferable where questionnaire brevity is an issue. From a public health perspective, recognizing that the public may not show expected “rational” behaviors during RIDEs is important. Therefore, risk probabilities alone are unlikely to be sufficient to motivate protective behaviors. What affective strategies to use to best motivate behavioral change awaits clarification.

## Competing interests

The authors declare that they have no competing interests.

## Authors’ contributions

QL participated in the study design, analyzed the data, interpreted the data and drafted the manuscript. BJC supervised the research, contributed to study design, data interpretation and amended the manuscript. WWTL contributed to study design, data interpretation and amended the manuscript. DWMN contributed to questionnaire design, coordinated data collection and amended the manuscript. RF conceived of the study, designed the questionnaire, interpreted data and amended the manuscript. All authors read and approved the final manuscript.

## Pre-publication history

The pre-publication history for this paper can be accessed here:

http://www.biomedcentral.com/1471-2334/14/169/prepub

## Supplementary Material

Additional file 1: Figure S1 The A/H1N1 pandemic curve in Hong Kong and timeline of the surveys. Click here for file

Additional file 2: Table S1 Questions for measuring anxiety, worry and risk perception in the study and their associated response scales. Click here for file
