# Supplementary figures and images for: Anxiety, worry and cognitive risk estimate in relation to protective behaviors during the 2009 influenza A/H1N1 pandemic in Hong Kong: ten cross-sectional surveys
Source: BMC Infect Dis. 2014 Mar 27;14:169. doi: 10.1186/1471-2334-14-169 (PMC3986671; doi:10.1186/1471-2334-14-169)

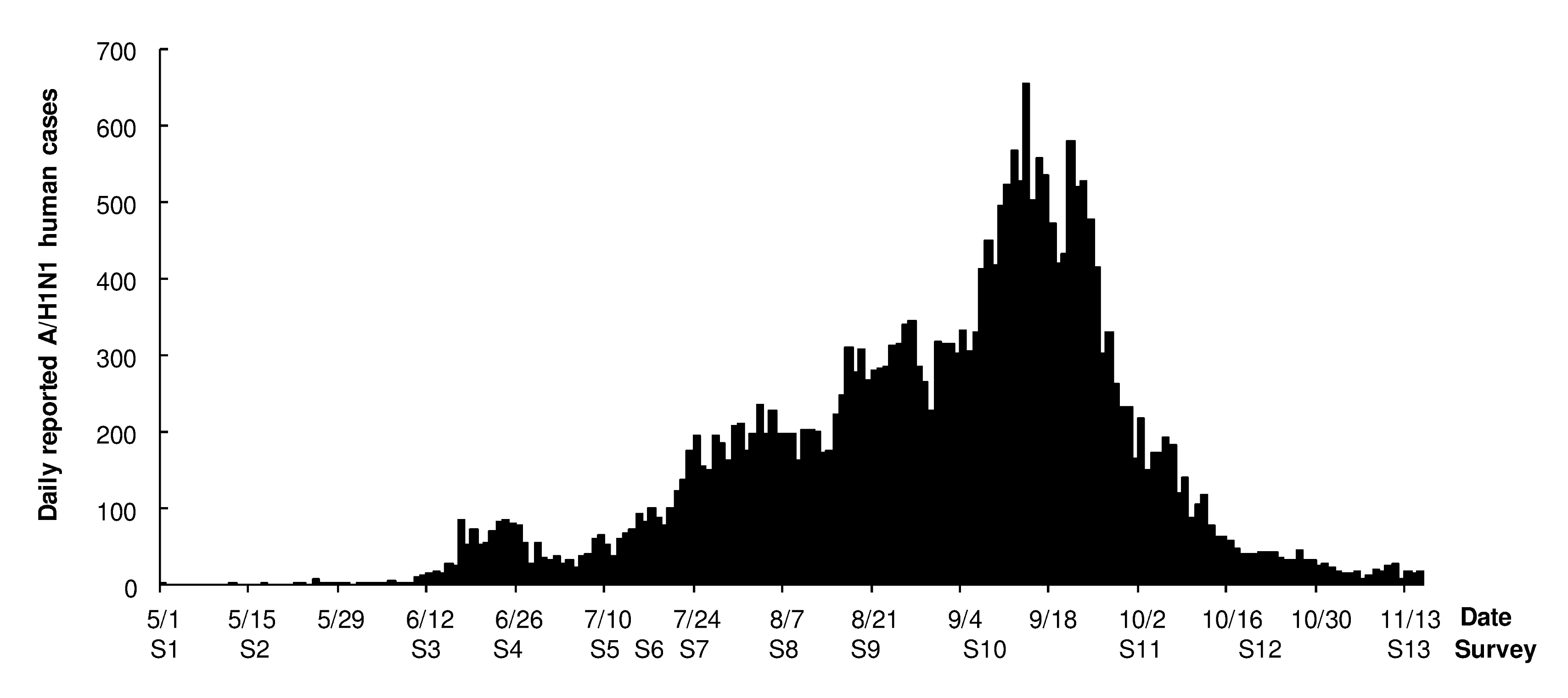

Supplement: Additional file 1: Figure S1 — The A/H1N1 pandemic curve in Hong Kong and timeline of the surveys. [file 1471-2334-14-169-S1.tiff]
