# Supplementary material for: Anxiety, worry and cognitive risk estimate in relation to protective behaviors during the 2009 influenza A/H1N1 pandemic in Hong Kong: ten cross-sectional surveys
Source: BMC Infect Dis. 2014 Mar 27;14:169. doi: 10.1186/1471-2334-14-169 (PMC3986671; doi:10.1186/1471-2334-14-169)
Supplement: Additional file 2: Table S1 — Questions for measuring anxiety, worry and risk perception in the study and their associated response scales. [file 1471-2334-14-169-S2.docx]

Additional file 1: Table S1

| Measures | Items | Response scale | Dichotomous scale |
| --- | --- | --- | --- |
| Anxiety | Agreement on ten statements:  I feel rested / content / comfortable / relaxed / pleasant / anxious / nervous / jittery / "high strung" / over-excited and "rattled" | 1=not at all, 2=sometimes, 3=Moderately so, 4=very much so | 1= high anxiety, mean score of 2.5-4.0, 0= low-medium anxiety, mean score of 1.0-2.4 |
| Anticipated worry | If you were to develop flu-like symptoms tomorrow, would you be | 1=not at all worried, 2=much less worried than normal, 3=worried less than normal, 4=about the same, 5=worried more than normal, 6=worried much more than normal, 7=extremely worried | 1= "worry more/much more than normal or extremely worry", 0= "worry about the same/less/much less than normal/not at all worried" |
| Experienced worry | In the past one week, have you ever worried about catching influenza A/H1N1? | 1=no, never think about it, 2=think about it but it didn't worry me, 3=worried me a bit, 4=worried me a lot, 5=worried about it all the time | 1="worry a bit/a lot/all the time", 0= "never think about it/ think about it but not worried" |
| Current worry | Please rate the current level of your worry towards human swine flu: | Score ranged from 1-10 (1 =very mild, 10 =very severe) | 1= level of 6-10  0= level of 1-5 |
| Perceived absolute susceptibility to A/H1N1 | How likely do you think it is that you will contract swine flu (H1N1 influenza A) over the next 1 month? | 1=Never, 2=very unlikely, 3=unlikely, 4=evens, 5=likely, 6=very likely, 7=certain | 1= "likely/very likely/ certain", 0= "never/ very unlikely/ unlikely/ evens" |
| Perceived relative susceptibility to A/H1N1 | What do you think are your chances of getting swine flu (H1N1 influenza A) over the next 1 month compared to others outside your family?^#^ | 1=not at all, 2=much less, 3=less, 4=evens, 5=more, 6=much more, 7=certain | 1= "more/ much more / certain", 0= "not at all/ much less/ less/ evens" |
| Perceived A/H1N1 severity relative to SARS | How does swine flu (H1N1 influenza A) compare with SARS in terms of seriousness? | 1=much less, 2=less, 3=about the same, 4=more, 5=much more | 1= "more/much more", 0= "much less/ less/ about the same" |
| Perceived A/H1N1 infectivity relative to seasonal flu | Please compare human swine flu with seasonal flu. Do you think the infectivity rate of swine flu is: | 1=much higher than seasonal flu, 2=a little higher than seasonal flu, 3=same as seasonal flu, 4=a little lower than seasonal flu, 5=much lower than seasonal flu | 1= "much/a litter higher than seasonal flu", 0="same/a little low/much lower than seasonal flu" |
| Avoiding crowded places | In the past seven days, did you avoid going to crowded places? | 1=yes, due to swine flu, 2=yes, but not due to swine flu, 3=no | 1= "yes, due to swine flu",  0= "yes, but not due to swine flu or no" |
| Maintaining good indoor ventilation | In the past seven days, did you keep good indoor ventilation? | 1=yes, due to swine flu, 2=yes, but not due to swine flu, 3=no | 1= "yes, due to swine flu",  0= "yes, but not due to swine flu or no" |
| Disinfecting household frequently | In the past seven days, did you clean or disinfect house more often? | 1=yes, due to swine flu, 2=yes, but not due to swine flu, 3=no | 1= "yes, due to swine flu",  0= "yes, but not due to swine flu or no" |

^#^ It was revised to be "what do you think are your chances of getting swine flu (H1N1 influenza A) over the next 1 month compared to other people outside your family of a similar age?" from Survey 10 to Survey 13.
